# Silicon rich nitride: a platform for controllable structural colors

**DOI:** 10.1515/nanoph-2024-0454

**Published:** 2024-10-31

**Authors:** Oren Goldberg, Noa Mazurski, Uriel Levy

**Affiliations:** The Faculty of Science, The Center for Nanoscience and Nanotechnology, Institute of Applied Physics, 26742 The Hebrew University of Jerusalem, Jerusalem 91904, Israel; Singapore-HUJ Alliance for Research and Enterprise (SHARE), The Smart Grippers for Soft Robotics (SGSR) Programme, Campus for Research Excellence and Technological Enterprise (CREATE), Singapore 138602, Singapore

**Keywords:** dielectric metasurface, silicon rich nitride, structural colors

## Abstract

High refractive index dielectric materials like silicon rich nitride (SRN) are critical for constructing advanced dielectric metasurfaces but are limited by transparency and complementary metal oxide semiconductor (CMOS) process compatibility. SRN’s refractive index can be adjusted by varying the silicon to nitride ratio, although this increases absorption, particularly in the blue spectrum. Dielectric metasurfaces, which utilize the material’s high dielectric constant and nano-resonator geometry, experience loss amplification due to resonance, affecting light reflection, light transmission, and quality factor. This study explores the impact of varying the silicon ratio on structural color applications in metasurfaces, using metrics such as gamut coverage, saturation, and reflection amplitude. We found that a higher SRN ratio enhances these metrics, making it ideal for producing vivid structural colors. Our results show that SRN can produce a color spectrum covering up to 166 % of the sRGB space and a resolution of 38,000 dots per inch. Fabricated samples vividly displayed a parrot, a flower, and a rainbow, illustrating SRN’s potential for high-resolution applications. We also show that SRN can provide a better CIE diagram coverage than other popular metasurfaces materials. These findings highlight the advantages of SRN for photonic devices, suggesting pathways for further material and application development.

## 1 Introduction

The advancement in precise nano-fabrication processes and computational power over the past couple of decades has opened a new research avenue in the field of nanophotonics. The ability to calculate the electromagnetic fields within nanoscale devices and to precisely fabricate these nanoscale structures has led to the realization of structures that do not adhere to classical optical behavior due to their small features (subwavelength in size). These structures are often considered as a material with locally varying optical constants (i.e., dielectric constant, birefringence, dispersion) that are highly dependent on the geometry of the single nanostructure. The two-dimensional version of such materials is now known as a “metasurface”, which is comprised of subwavelength structures arranged in an array placed on the surface of a substrate [1]. By controlling the geometry size, symmetry, periodicity, and orientation of the nanostructures one can control the properties of light (e.g. amplitude, phase, spectral response, polarization and more) and manipulate the light matter interactions such that a tailored response from the optical system is achieved [2], [3].

Typically, one would differentiate between two types of metasurfaces: plasmonic/metallic and dielectric. Plasmonic metasurfaces are comprised of metallic structures that support localized surface plasmon resonances in the form of excited free electrons that are coupled to the electromagnetic wave and oscillate in the confined metal\dielectric interface [3]. These types of metasurfaces have the advantage of strong enhancement of the near field, yet their performance are limited due to the inherently Ohmic losses in the metal [4], [5] and due to their inability to support transverse magnetic (TM) like modes (due to boundary condition symmetry) [6]. On the other hand, high-index dielectric metasurfaces that operate below the photon absorption energy have low losses and can support both TM and transverse electric (TE) modes [7]. These modes form when an external radiation is coupled to subwavelength dielectric structures (with sufficiently large refractive index) and excite density currents of the bound electrons within the dielectric material [8]. These currents may traverse various pathways within the structure, generating a local electric or magnetic multipolar field. Each multipole represents a resonance of the structure, commonly known as a Mie-type resonance [7], [8].

A major limitation of dielectric metasurfaces lies in the limited selection of dielectric materials available for use which have both a high enough refractive index (typically *n* > 2) and do not absorb in the spectral region of interest [9]. For example, for the IR and NIR regime silicon is a favourable material for use as it has a refractive index of ∼3.6 and negligible absorption [5], [10], [11], [12], [13], [14], [15]. Yet, although possible, it is more challenging to use silicon for the visible region due to its strong absorption [16], [17], [18], [19]. As a result, two major materials used in the visible region are titanium dioxide and silicon nitride which have refractive index of ∼2.4 and ∼2, respectively, and both have negligible loss in the visible range [20], [21]. Each material has its own advantages and disadvantages. For instance, silicon nitride is considered as complementary metal oxide semiconductor (CMOS) compatible [22] but has a relatively low refractive index when compared to other high index materials, which means that inherently the structures are larger in their footprint and are limited in the achievable field confinement due to the relatively low contrast of the refractive index compared to the surroundings. On the other hand, titanium dioxide has a higher refractive index but is not CMOS compatible and is typically more difficult to work with, meaning it is not industry ready. Silicon rich nitride (SRN) has recently surfaced in the field of dielectric metasurfaces as a viable alternate high-index dielectric material which could operate in the visible regime [23], [24], [25], [26]. Interestingly, by increasing the amount of silicon in silicon-nitride beyond the stoichiometric ratio it is possible to increase the refractive index of the material, albeit with an addition of a small loss, mostly in the blue part of the optical spectrum. As such, controlling the amount of silicon in SRN introduces a tunability feature which provides an additional important free parameter for designing dielectric metasurfaces.

As many metasurfaces are implemented as resonant structures, any loss introduced into the system may be amplified and most likely degrade the performance of the metasurface. As such, a question arises regarding the use of lossy materials for resonant nano structures. On the one hand, to achieve enhanced scattering cross section and maintaining structures with low aspect ratio (height to width ratio), a large refractive index is desired [8]. On the other hand, due to the strong confinement of the optical mode in the nano structure, the total loss might be enhanced, jeopardizing the performance of the device (e.g. amplitude of reflection/transmission). An effective way to further explore this notion is to implement a metasurface which supports structural colors. Structural colors are colors that appear due to the scattering of light off a patterned nanostructure or the filter response of stacked dielectric layers, unlike pigments or dyes which produce color by absorbing light. Structural colors have been demonstrated in both plasmonic [27], [28], [29], [30], [31], [32], [33], [34] and dielectric [17], [27], [33], [35], [36], [37], [38], [39], [40], [41], [42], [43], [44], [45] metasurfaces. In addition to producing color, structural colors could be used for spectral filtering [46], colorimetric sensing [47], and data archival [18]. Utilizing this platform, the significant impact of the dielectric material loss will become evident, allowing for precise conclusions.

Thus, in this work we use SRN dielectric metasurfaces which on top of being a CMOS compatible material, supports Mie-type resonances, specifically magnetic dipole (MD), acting as spectral filters resulting in the demonstration of structural colors. We compare the performances of the several SRN ratios and provide a comprehensive look at the advantages and disadvantages of using dielectric materials with a small loss for resonant structures. Finally, we demonstrate the usefulness of SRN with high ratio of silicon to nitride in producing high resolution and high-quality colorful images.

## 2 Results and discussion

Depositing SRN thin films is done by plasma enhanced chemical vapor deposition (PECVD) method [48], [49]. By manipulating the ratio of the reactant gasses used in the PECVD process one can control the refractive index of the SRN thin film. The gasses used in the process are SiH_4_ and NH_3_ and we denote the ratio between the atomic concentration of silicon and nitride as *β* = Si/N. It can be seen in Figure S1 that as *β* increases so does the refractive index and the extinction coefficient (which is the imaginary part of the complex refractive index and is correlated to the loss of the material). Overall, all ratios have negligible loss from wavelength of 600 nm onwards. Figure S2 shows the correlation between the atomic concentration ratio to the reactant gas ratio used in the process.

**Figure 1:**
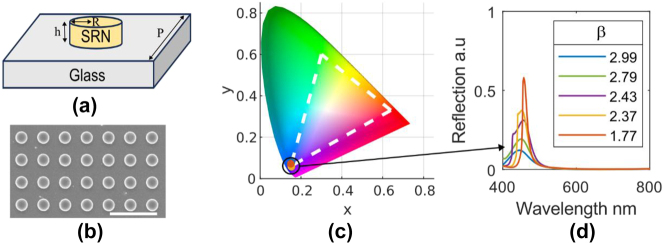
Structural design of unit cell and optical response of SRN metasurface. a) A schematic of the metasurface unit cell. The unit cell consists of an SRN disk with radius *R* and thickness of *h* = 135 nm. The period is determined by the relation of *P* = 2*R* + gap, where the gap is chosen to be 130 nm. b) SEM image of a fabricated sample metasurface. Scale bar (white) is 1 μm. c) CIE 1931 chromaticity diagram. The five overlapping points on the diagram correspond to the five reflection spectra seen in (d). d) Simulated reflection spectra (absolute reflection from the metasurface) of five different values of SRN and different unit cell geometries. The radii are *R* = 60, 70, 80, 85, 90 nm which correspond to *β* = 2.99, 2.79, 2.43, 2.37, 1.77 respectively. All unit cells have the same thickness.

We designed a dielectric metasurface which acts as a spectral filter in reflection to achieve structural colors and to compare the performances of the different SRN ratios. This method was chosen due to its ability to visibly demonstrate the difference in performance. As a starting point we optimized the unit cell structure for all *β* values such that the reflection spectra of the metasurface will translate to a point on the bottom left corner on the sRGB diagram (blue) as seen in Figure 1c due the nature of all the materials having non-negligible loss in that spectral region. The points in Figure 1c are attained from transforming (using color matching functions as seen in the Supplementary material S8) simulated reflection spectra which appear in Figure 1d.

The metasurface consists of an SRN disk with a thickness of 135 nm (in depth analysis for thickness choice can be seen in the Supplementary material S9), on top of a glass substrate. The unit cell upholds a relation between the radius and the period such that the gap between adjacent disks remains constant. In our work the gap was chosen to be 130 nm. An illustration of the unit cell is seen in Figure 1a and b shows a SEM image of a fabricated metasurface. Independent reflection simulations were run to find the optimal geometric parameters for each *β* value such that they will land in the desired point on the chromaticity diagram. We fixed the thickness of all the unit cell geometries to 135 nm. The radii for each unit cell were different and equal to *R* = 60, 70, 80, 85, 90 nm (from largest value of *β* to lowest).

By design, all the structures occupy the same space on the CIE 1931 diagram which means that their center wavelengths and full width half max (FWHM) values are very similar (up to small variations) as seen in Figure 1d. On the other hand, the amplitude of the reflection spectra varies and decreases as the value of *β* increases, which means that the structures with the lower index value will appear brighter than the ones with higher index. This is due to the enhanced absorption of the higher index structures. The dominant optical mode supported by the metasurface structure is the MD mode, a cross-section of the magnetic field distribution in the disk can be seen in Figure S3. As can be seen, the magnetic field becomes more confined within the nano disk structure as *β* increases resulting in a larger overlap between the optical mode and the absorbing material, contributing to the enhancement of the absorption. In addition, higher values of *β* have a larger extinction coefficient for this spectral range which inherently increases the absorption further.

**Figure 2:**
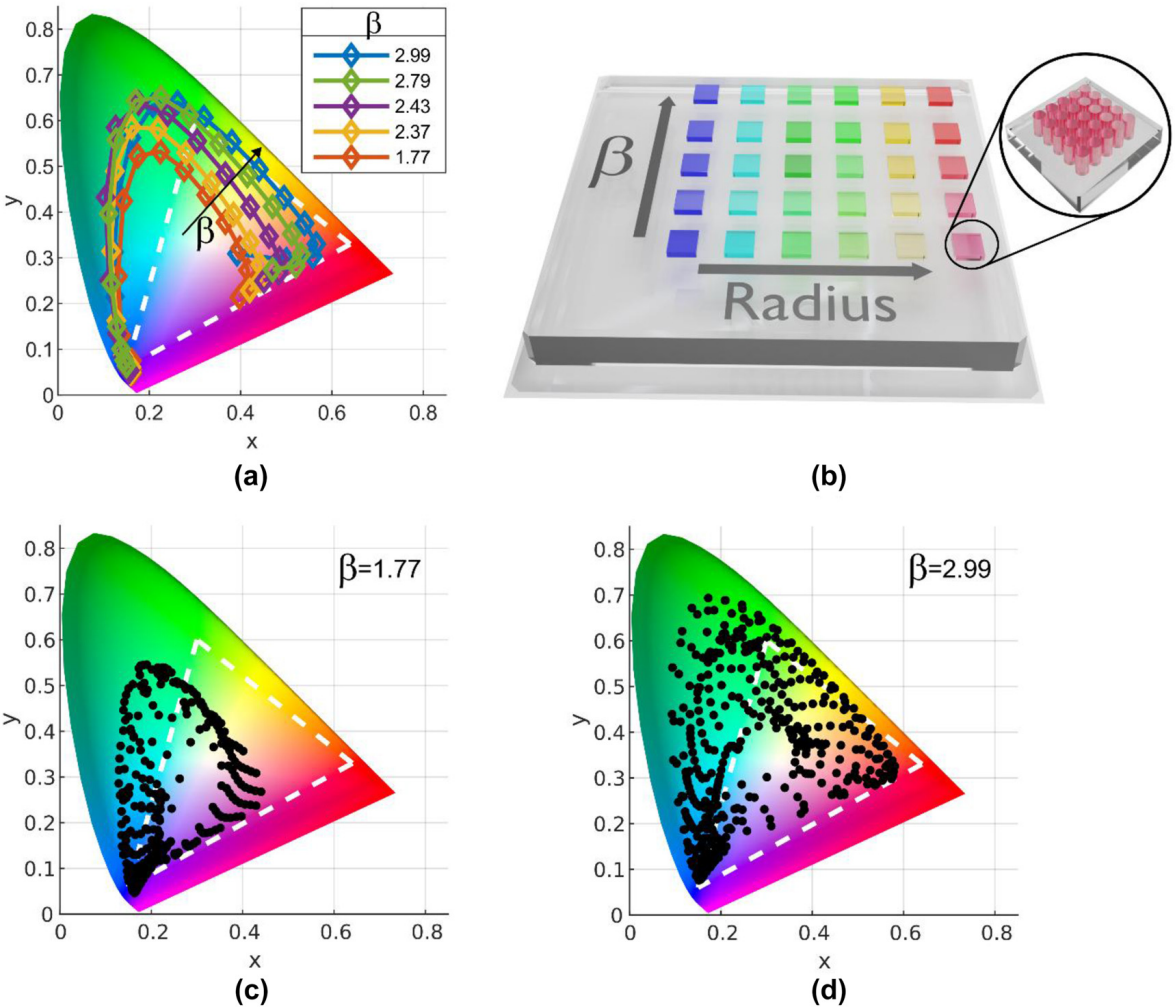
Color gamut and chromaticity analysis of SRN metasurfaces for varied geometries. a) Chromaticity diagram calculated for different *β* values for arrays with a thickness of *h* = 135 nm, *R* = 70–150 nm and *P* = 270–430 nm with a constant gap of 130 nm. For wavelengths shorter than ∼550 nm all curves are mostly overlapping (indicating that they are generating the same color regardless of the different refractive index). In this region the loss is non-negligible. For longer wavelengths, the response is more dispersive. As *β* decreases so does the gamut. The white triangle denotes the sRGB region. b) An artistic illustration of (a) visualizing the different color ranges that each SRN ratio can cover for the same geometry. c, d) SRN1.77 and SRN2.99 respectively. A nested sweep of the radius and gap has been performed which is represented by the black dots in the diagrams. For each value of *R*, which ranges from 80–150 nm, the gap was varied from 10 to 190 nm. Both instances cover a large area in the diagram where (c) covers 87 % of the sRGB and (d) 166 %.

The metric chosen for comparison between the different SRN ratio metasurfaces is the coverage area of the CIE 1931 chromaticity diagram. We use the optimized structures from Figure 1c as a base line for comparison. Increasing the radius and keeping the gap between adjacent disks constant results in a spectral shift of the reflection spectra. This shift is translated into translation of the points (seen in Figure 1c) on the chromaticity diagram, the area encompassed by the points represent all the colors which could be generated by the metasurface. The radius varies from 70 to 150 nm upholding the relation of *P* = 2*R* + 130 nm with a constant height of *h* = 135 nm. Figure 2a shows the calculated translation of the points of the five different *β* values on the chromaticity diagram.

We see that as *β* increases so does the area covered in the chromaticity diagram, indicating that the performances of the structural color metasurfaces with higher refractive index are better. Furthermore, the routes described by the points branch out after reaching a wavelength of ∼550 nm which coincides with the wavelength at which the loss of the SRN becomes negligible for all ratios. This point helps to understand the role of the loss and how it is affecting the overall performance of the metasurface. The loss of the material causes a widening of the resonance in addition to damping of the amplitude. This can be seen clearly in Figures S4 and S5a where the FWHM decreases with the radius and the amplitude increases. When the loss becomes negligible, the resonance sharpens, resulting in a reduction of the saturation of the color generated by the metasurface, which is why the routes of the lower *β* values curve into the middle of the chromaticity diagram where the saturation is low. On the other hand, the higher index metasurfaces have a larger saturation pushing them towards the edges of the diagram allowing them to cover a larger area. Figure 2b illustrates the behavior described so far.

The choice of the optimal *β* value to be used depends on the functionality of the final device. For instance, for the purpose of refractometry (which is used for identifying biological substances) a lower value of *β* is desirable due to its higher quality factor and the lower confinement of the MD mode to the nano disk (compared the higher *β* values) making it more sensitive to changes in the surrounding medium. For coloring and information storage a higher *β* value is desired as it can cover a large area in the CIE 1931 chromaticity diagram, meaning that a wide range of colors can be achieved, albeit at the expense of a lower brightness for blue colors. In this work the focus is on the coloration capabilities of the material, therefore the emphasis is on the gamut coverage. Thus, a comparison between the two extreme SRN ratio materials is conducted. A nested sweep is performed on the *β* = 1.77 and 2.99 structures with a constant height of 135 nm. For every radius value (ranges from 80 to 150 nm) the gap varies from 10 to 190 nm. The corresponding gamut coverage is seen in Figure 2c, d and shows that SRN1.77 covers 87 % of the sRGB whereas the SRN2.99 covers a much larger area, 166 % of the sRGB. So clearly, a higher value of *β* assists in obtaining larger coverage of the CIE 1931 chromaticity diagram. Yet, while not shown on the diagram, the reflection for the blue colors decreases with the increase in *β*, and thus the brightness of the blue is reduced. For low light applications, where brightness becomes important, this is a parameter to be considered. The gamut coverage of several common high dielectric materials which are frequently used in the field of metasurfaces (such as TiO_2_, GaN) is shown in Figure S7 as a comparison to the gamut achieved by SRN2.99. We see that the lower index materials have a similar coverage to the lower *β* values of SRN indicating that SRN2.99 is still a better choice for coloration under the optimization conditions we chose to work with.

**Figure 3:**
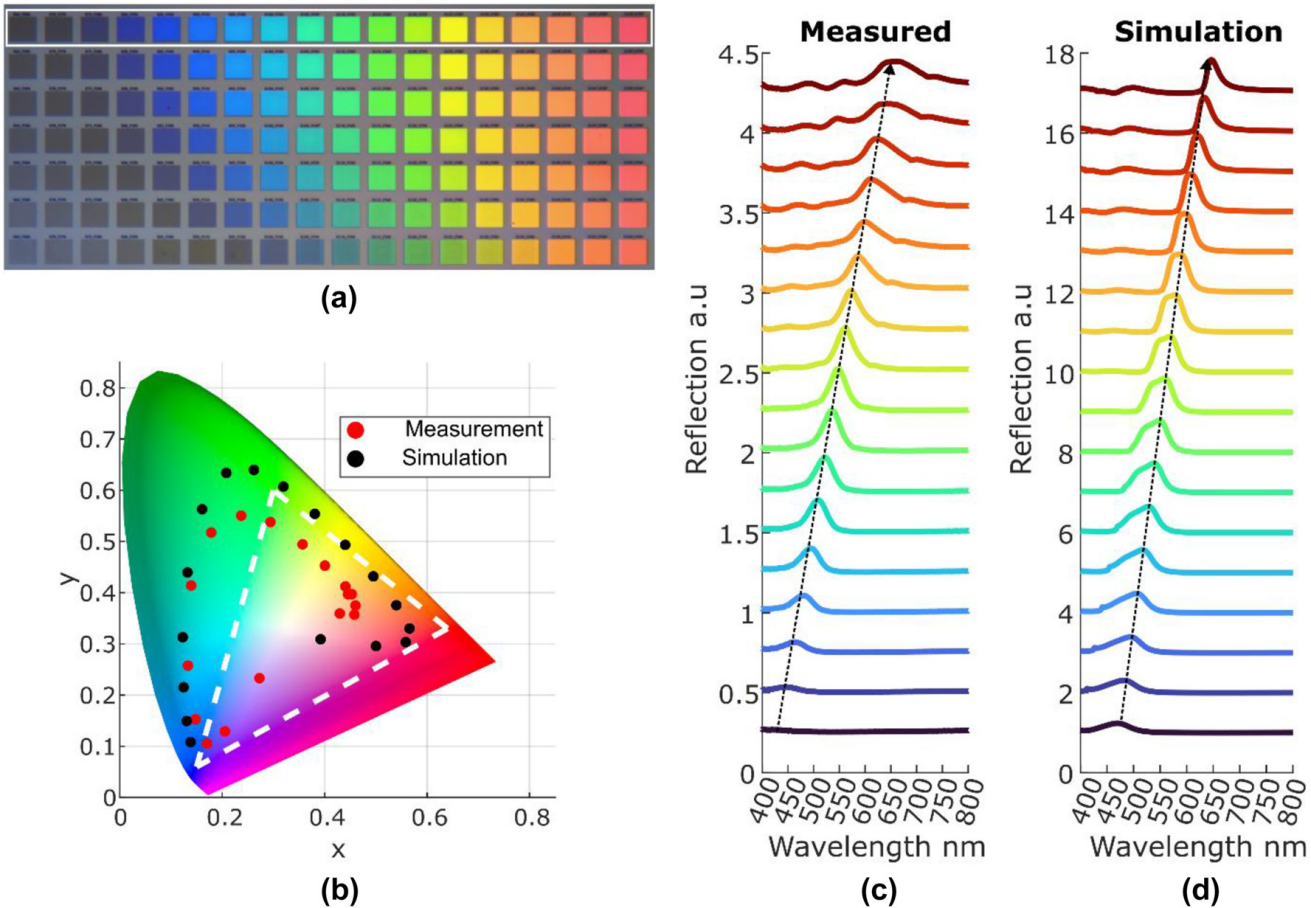
Microscopic imaging and reflection spectra comparison of SRN metasurface arrays. a) An image taken from and optical microscope of a fabricated samples using SRN with *β* = 2.99. b) A comparison between measured and simulated results from c and d. c, d) measured and simulated reflection spectra of the arrays from (a) marked in white rectangle. Spectral tuning of the resonance is achieved by increasing the radius and keeping the gap constant, resulting in a wide coverage of the chromaticity diagram. An offset of 0.5 and 1 is added to (c) and (d) respectively for visual purposes.

Following the simulation results, metasurface samples based on SRN2.99 film were fabricated to showcase the capabilities of the material to produce vivid structural colors. The high *β* film was chosen as this ratio gives the largest coverage of the CIE 1931 chromaticity diagram. Using the values extracted from the simulation results (Figure 2a) several arrays of nano disks were fabricated. First, a dose test is performed to find the optimal conditions for obtaining the desired geometrical features as seen in the optical microscope image in Figure 3a. Figure 3c and d showcase the measured and simulated reflection spectra of the metasurfaces related to the first row of Figure 3a (marked by the white box). All optical measurements were performed with unpolarized light as the metasurface design is symmetrical to 90° rotations. Simulations and measurements are in good agreement and they both showcase the ability to tune the spectrum over the whole visible range. Unfortunately, fabrication tolerances are unavoidable and play a role in lowering the performance of the fabricated metasurface compared to the simulated counterpart. And indeed, focused ion beam (FIB) measurements (seen in Figure S6A–B) show that there is a 50 nm over etch region into the glass substrate, a slight angle of ∼5° to the sidewalls of the disk and some additional roughness to them which results in unwanted scattering. This causes an overall reduction in reflection amplitude and for the larger radii a substantial widening of the resonance. This is further visualized in Figure 3b where it is seen that the measured values curve into the middle of the chromaticity diagram unlike the simulated values. Thus, there is some difference between the gamut coverage where in simulation 128 % of sRGB is covered whereas the measured devices cover 79 % of the sRGB. Further optimization of the fabrication process is expected to push the measured results closer to the simulation conditions as can be seen in Figure S6C where it is apparent that not all the radii can be fabricated under the same conditions (smaller radii show a tendency to have angled side walls compared to larger radii that do not). Another reason for the small discrepancy between simulation and experimental results arises from the measurement method. As described in the methods section, both spectrum and imaging measurements used objectives to focus the light on the fabricated samples, meaning they we’re not illuminated at normal incidence. As seen in Figure S11, increasing the angle of incidence will result in a slight red shift of the reflection spectrum in addition to reducing the reflection amplitude. This will cause a widening of the reflection spectrum which can be seen in Figure 3C. Thus, changing the optical setup to illuminate with normal incidence will improve the measured results.

**Figure 4:**
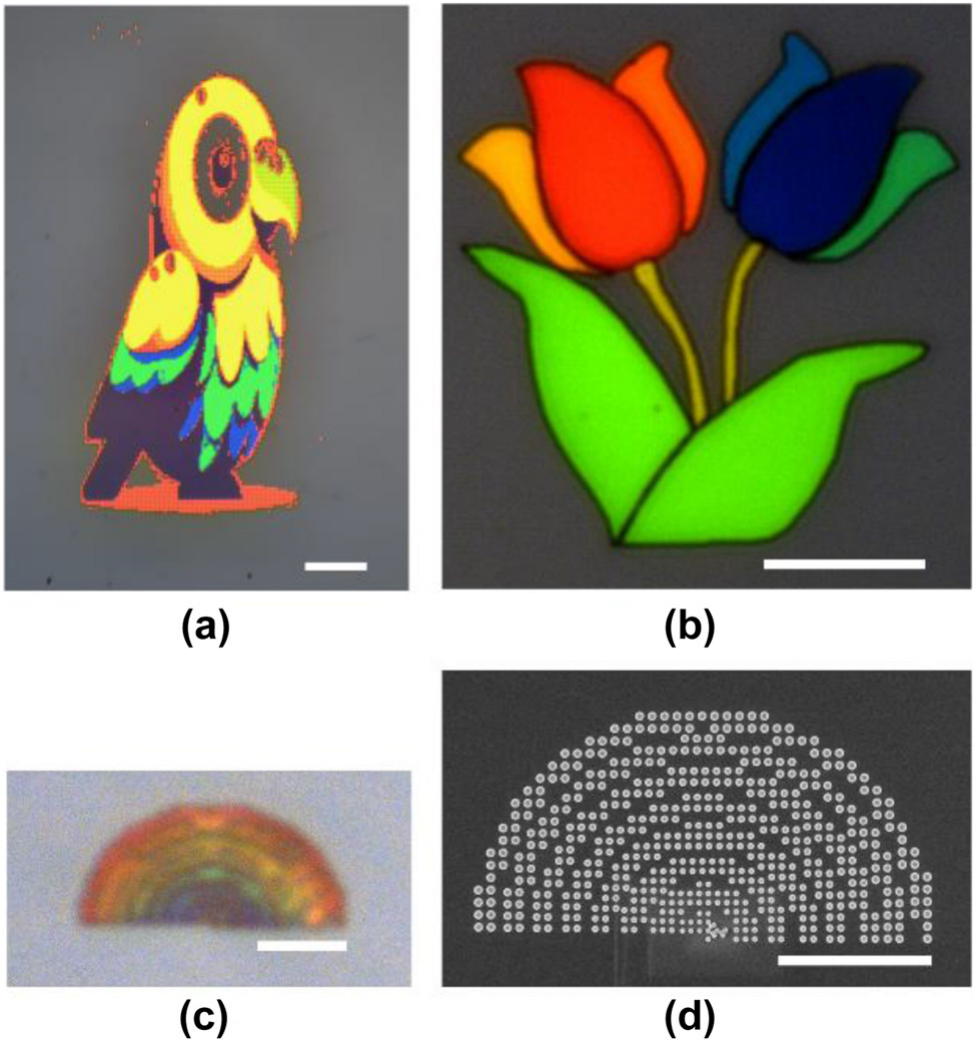
Fabricated pictures of parrot tulips and a rainbow demonstrating the capabilities of the structural color metasurface taken via an optical microscope. a) Pixelated images of a parrot (image was generated using AI software DALL-E). Each pixel is 5 × 5 μm^2^ and the scale is 100 μm. b) A tulip picture constructed using eight different color values (the image was drawn by hand). The two bottom leaves and the two stems have the same color, whereas each petal of the flower has a different color. Each region of the flower is constructed of continuous array. The scale bar is 30 μm. c) A picture of a rainbow constructed using concentric rings with continuous arrays, scale bar is 5 μm. The colors generated are still visible and distinguishable between different regions of the rainbow, demonstrating the high resolution achievable using SRN. d) SEM image of the rainbow in (c), each arc is constructed of one or two nano disks, scale bar is 5 μm.

To demonstrate the high capabilities of SRN in producing high resolution and colorful images several samples were fabricated as seen in Figure 4. The first image is of a parrot generated using an AI software (DALL-E), the image consists of 128 × 256 pixels where each pixel 5 × 5 μm^2^ area consisting of the above mentioned metasurface, resulting in a total size of the image being approximately 640 × 1,280 μm^2^. The color of each pixel was determined by direct image matching, where the colors of the pixels in the original image were mapped to the colors which can be fabricated using SRN2.99 metasurfaces. As the original image color spectrum is limited to the sRGB region and the colors generated by the metasurface go beyond the sRGB region, matching between the two regions was done by taking the points with the closest distance on the chromaticity diagram. As can be seen (Figure 4a) a nicely looking parrot can be observed with a large span of vivid colors.

As explained, the parrot in Figure 4a is made of many metasurface arrays with varying radii and periods from one pixel to another, depending on the desired color. This means that there is a slight mismatch between similar and dissimilar adjacent pixels, as the pitch of the pixel is not an integer multiplication of the metasurfaces pitch which results in the image having a slightly pixelated look (not smooth). To overcome this limitation, two additional images with simpler features were fabricated. The fewer features allow for manual choice of the color in each region resulting in a continuous array design making the appearance of the image much smoother. These two images can be seen in Figure 4b and c, where the first one is of a tulip flower (original image drawn by hand) and the second one is of a rainbow. The tulip image showcases the vivid colors which could be generated using SRN and the rainbow indicates the high resolution achievable. Each arc of the rainbow is made up of one to two nano disks, as seen in Figure 4d, and the colors between each arc are distinguishable indicating that the resolution of the metasurface is of one period which translates approximately to 38,000 DPI.

## 3 Conclusions

In conclusion, we have demonstrated the importance of using materials with high refractive index for the demonstration of high-quality structural colors based on metasurfaces. Specifically, we have used SRN with different stoichiometric ratios to study the impact of increasing the refractive index of a dielectric material at the expense of a slight loss on the color quality and the ability to cover a large portion of the CIE 1931 color diagram. In particular, using dielectric metasurfaces which generate structural colors we compare the performance of several SRN ratios and using the gamut coverage as the comparison metric, we see that the gamut coverage increases with the increase in stoichiometric ratio where the lowest ratio value can cover 87 % of the sRGB and the highest ratio provides coverage as high as 166 % of the sRGB region in simulation. Following this clear trend, we have fabricated metasurface samples made of SRN films with the highest SRN ratio and experimentally observed gamut coverage of 79 % of the sRGB region. We further demonstrated the coloring capabilities by fabricating several images which demonstrate the vivid and high-quality colors that are obtained by our SRN based metasurface, with a high resolution of ∼38,000 DPI. We also show that SRN can provide a better coverage of the CIE diagram as compared with other popular materials for metasurfaces. We believe that SRN is an outstanding choice for creating cost effective and manufacturable high brightness and vivid structural colors. In the future, we will extend the capabilities of these structural colors towards a dynamic response based on strain, with myriad applications in display, sensors, and soft robotics, to name a few.

## 4 Methods

*Simulations:* Simulations were performed using the commercially available FDTD software (Ansys Lumerical). All simulations consist of a SRN nano disk with a height of 135 nm, and varying period and radius. The unit cell had a periodic boundary condition in the *x* and *y* axis and PML in the *z* axis. The unit cell was excited by a plane wave and the optical constants used for the SRN are taken from Figure S1a and b.

*Sample Preparations:* First a thin film of SRN is deposited using PECVD onto a glass substrate, the chamber is kept at a constant temperature of 300 °C and pressure of 2 Torr. The stoichiometric ratio is obtained by controlling the reactant gas ratio SiH_4_/NH_3_. Next the thin film undergoes O_2_ plasma treatment (Plasma Asher Diener, Pico) for 10 min to remove any moisture from the substrate surface before spin coating a thin layer (∼230 nm) of negative resist (maN-2403). An additional layer of e-spacer is spin coated to improve conduction on the resists surface. The device is then patterned using e-beam lithography (Elionix E-Beam Lithography System). After exposure the sample is rinsed in water for 1 min to remove the e-spacer, developed in AZ-726 for 40 s, rinsed again for 1 min in water and blown dried using N_2_. The sample is then dry etched by reactive ion etching using a recipe of CHF_3_ and SF_6_ (Corial ICP/RIE System) for 190 s and finally is cleaned with a second O_2_ plasma treatment for 10 min (Plasma Stripper, March, Jupiter) at a power of 100 W.

### 4.1 Optical measurements

*Reflection Spectra:* Using an inverted microscope Nikon Eclipse TE300, the sample is illuminated with a tungsten-halogen 100 W lamp. The light is focused on to the sample via a 50× objective (NA = 0.45). The same objective collects the reflected light which is then coupled to an Ocean Optics spectrometer (Flame T-XR1-ES) via a 50 mm doublet lens (ThorLabs AC254-050-AB) and multimode optical fiber (Ocean Insight QP600-2-VIS-NIR). A reference spectrum is taken using a ThorLabs mirror PF10-03-P0 and all reflection spectra acquired for the sample is normalized to this reference. Integration time was set to 100 ms, and averaging number of five.

*Optical Imaging:* Using an optical microscope Vickers Instruments Compound Binocular Microscope, the sample is illuminated with a halogen lamp. The light is focused on the sample via several objectives (VICKERS MICROPLAN 4× (NA = 0.1), OLYMPUS SLMPLN 20× (NA = 0.25), 50× (NA = 0.35)) and collected via the same objective. The reflected light is imaged on to a CMOS camera (ImagingSource DFK 33UX183).

*SEM and FIB Imaging:* SEM images were taken using an Extra-High resolution Scanning Electron Microscope Magellan 400 L (ThermoFisher). The image from Figure 1b was taken using a current of 6.3 pA and accelerating voltage of 2 kV. The image from Figure 4d was taken using a current of 25 pA and the same accelerating voltage as before. FIB samples were prepared using a Helios Nanolab 460F1Lite Dual Focused Ion Beam/scanning Electron Microscope (Thermofisher). The images are taken with a current of 10 pA, accelerating voltage of 1 kV and a tilt of 52°.

## Supplementary Material

Supplementary Material Details
