# Supplementary material for: Silicon rich nitride: a platform for controllable structural colors
Source: Nanophotonics. 2024 Oct 31;14(23):3911–9. doi: 10.1515/nanoph-2024-0454 (PMC12617834; doi:10.1515/nanoph-2024-0454)
Supplement: Supplementary file 1 — Supplementary Material Details [file j_nanoph-2024-0454_suppl_001.docx]

**Supporting information for "Silicon Rich Nitride: A Platform for Controllable Structural Colors"**

*Oren Goldberg*, Noa Mazurski and Uriel Levy**

Oren Goldberg, Noa Mazurski, and. Uriel Levy

Institute of Applied Physics, The Faculty of Science, The Center for Nanoscience and Nanotechnology

The Hebrew University of Jerusalem

Jerusalem 91904, Israel

Corresponding author: [Oren.goldberg@mail.huji.ac.il](mailto:Oren.goldberg@mail.huji.ac.il)


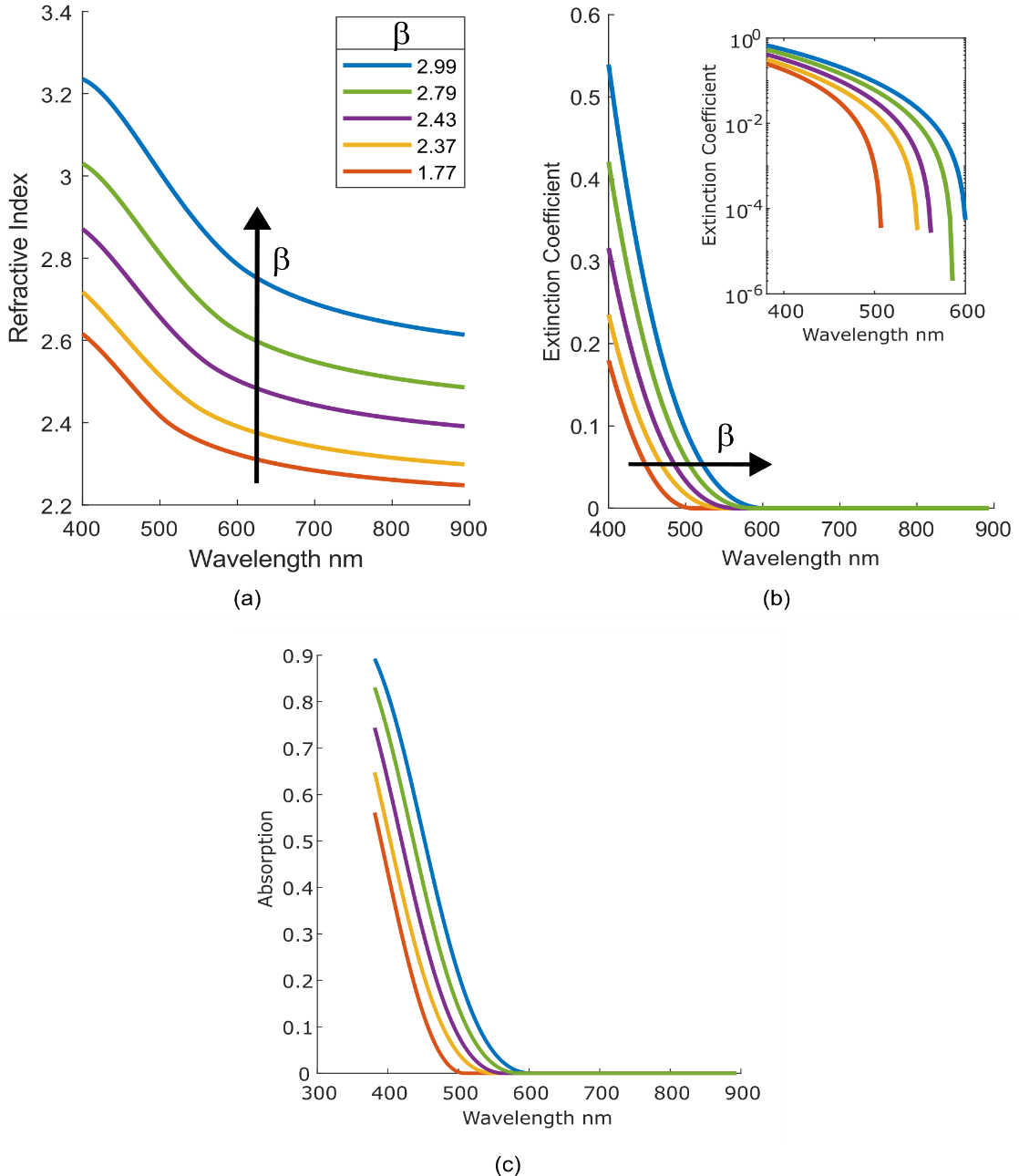


Figure S1: Silicon rich nitride optical constants measured via ellipsometry for five different gas ratios. a) Refractive index. b) Extinction coefficient. Inset is the representation in log scale. c) Absorption calculated using the values from (b), calculated by $A=1-e^{-\frac{4\pi\kappa}{\lambda_{0}}z}$, where z was taken to be 100nm.


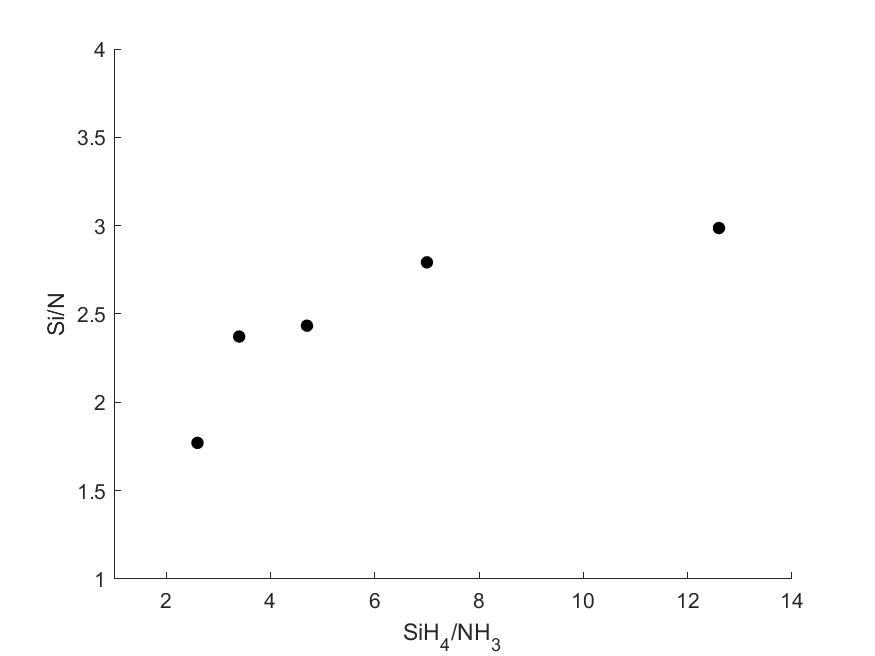


Figure S2: Atomic concentration ratio of silicon and nitride as a function of reactant gas ratio.


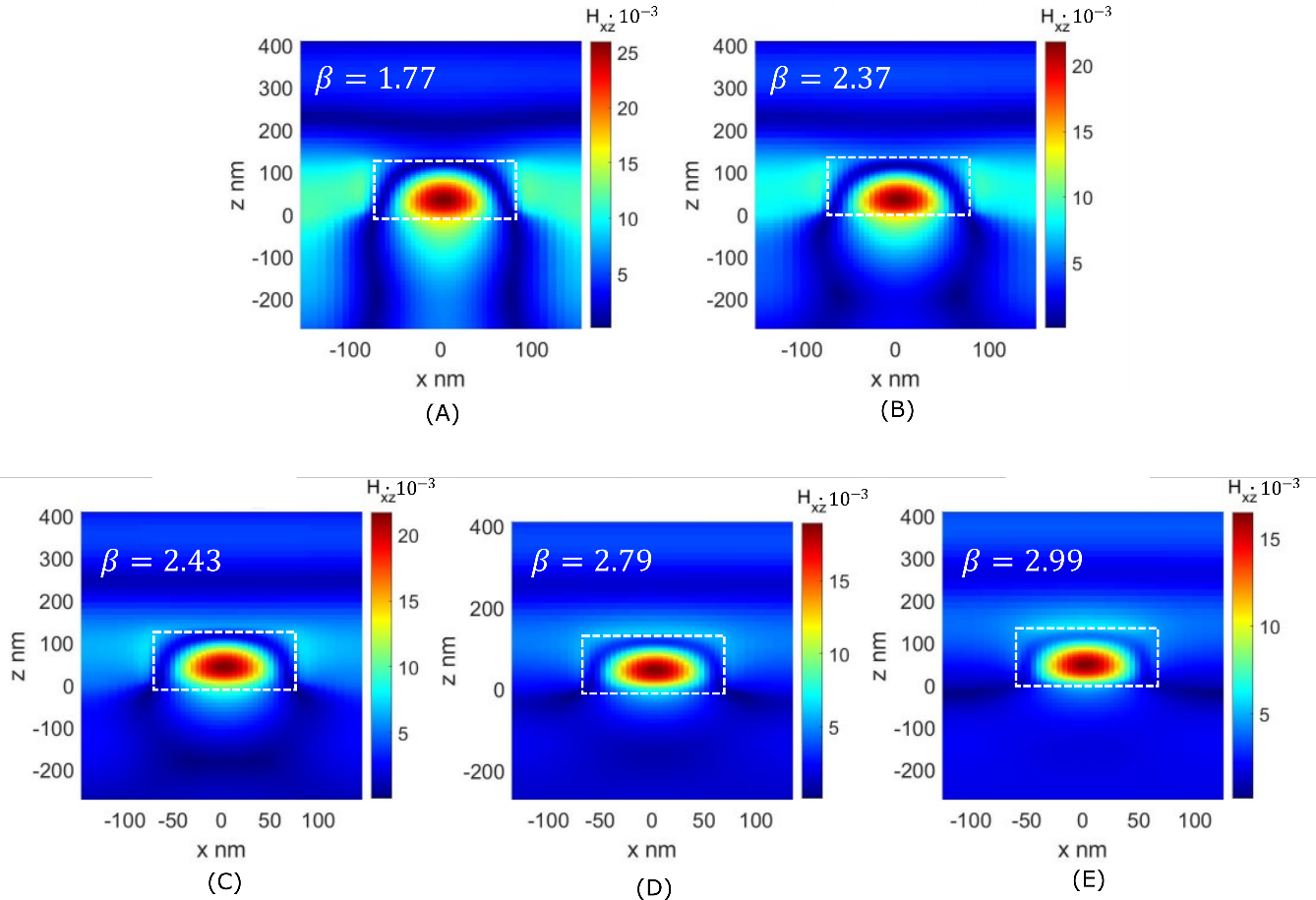


Figure S3: XZ cross section of the magnetic field in the nano disk structure for the resonant wavelength seen in the reflection spectra in Figure 4.D for the different SRN ratios. The wavelengths at which the magnetic fields are taken for the different SRN ratios $\beta=1.77, 2.37, 2.43, 2.79, 2.99$ are $457, 450, 455, 450 and$ 445nm respectively.


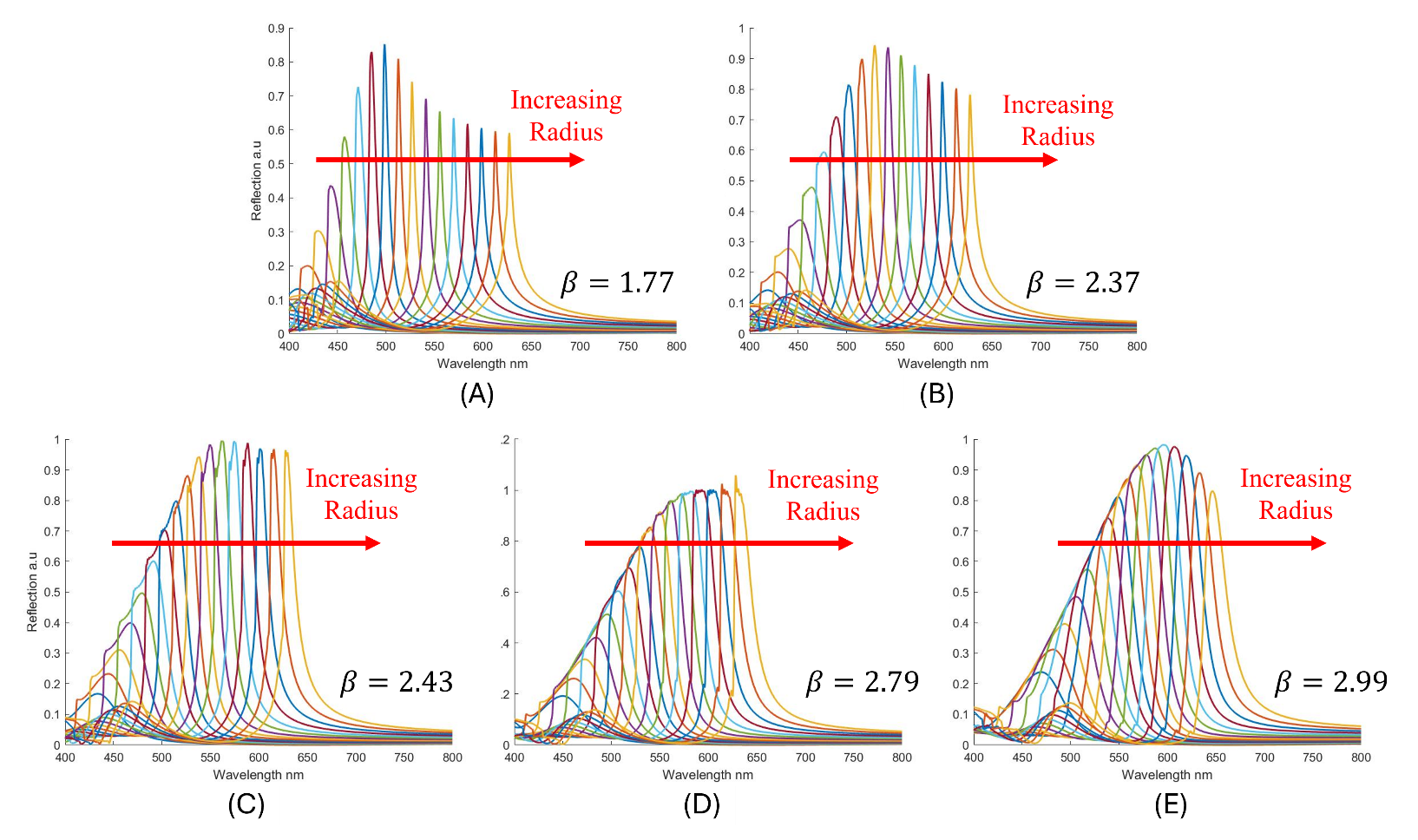


Figure S4: Reflection spectra of all five ratio SRN metasurface for a sweep of R=70-150nm, p=270-430nm and h=135nm (A total of 17 different geometries).


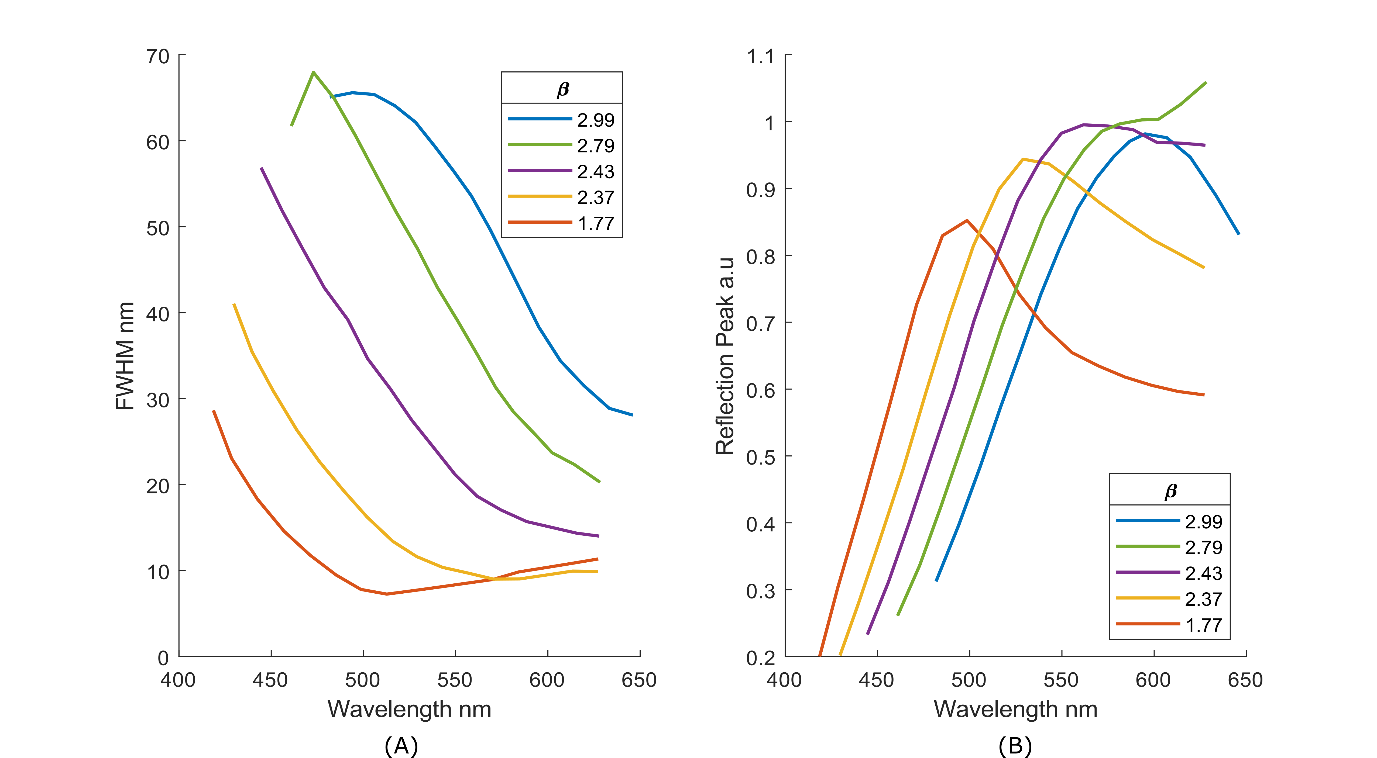


Figure S5: A) FWHM of the reflection spectra from S4. B) Reflection peaks of the reflection spectra from S4.


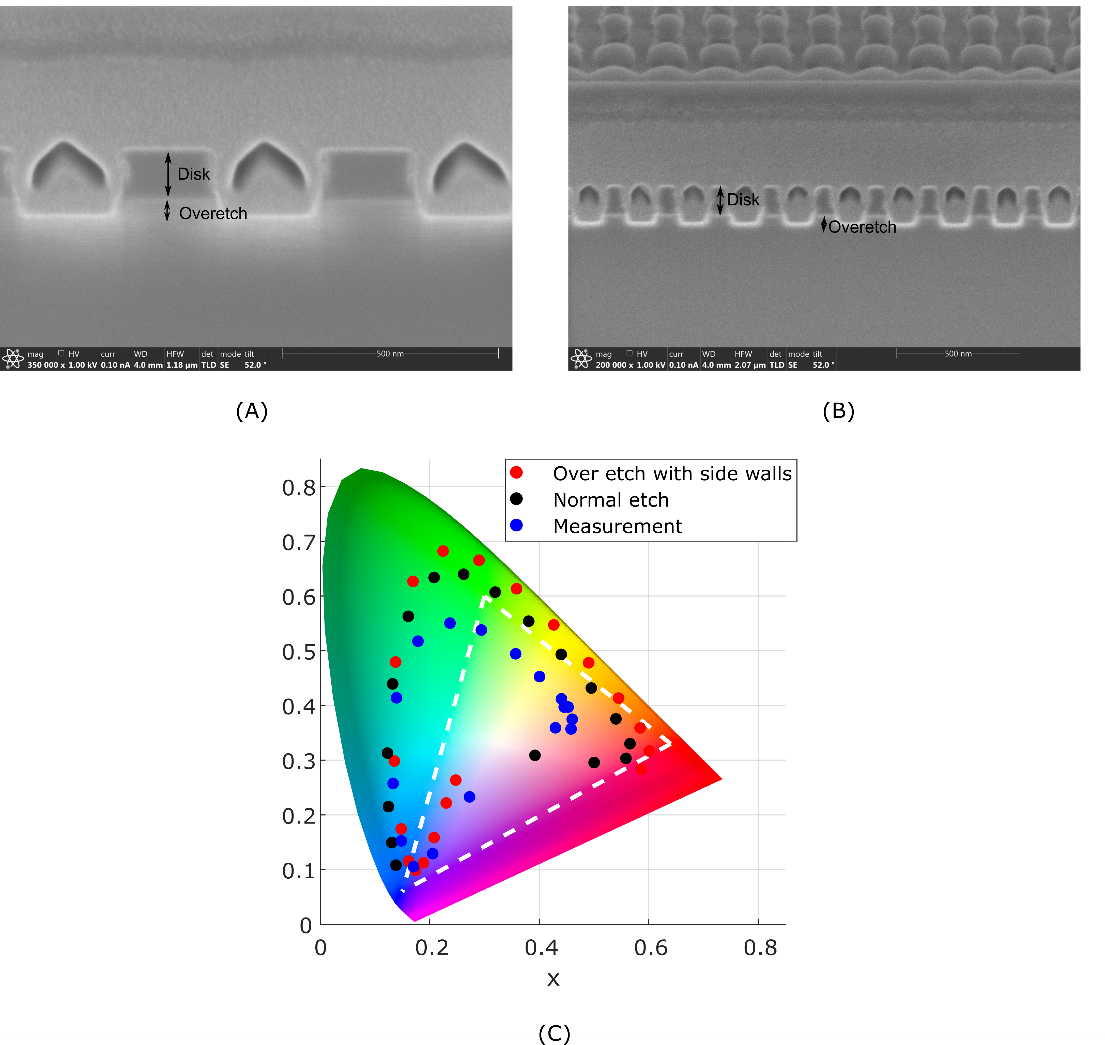


Figure S6: FIB cross section of fabricated SRN2.99 metasurface arrays. A) R=145nm and P=420nm. B) R=70nm and P=270nm. It is apparent that there is a 50nm over etching region into the substrate. In addition, a slight angle is apparent in the side walls of the fabricated disks for disks with small radii. C) A comparison of simulated reflection of desired design, simulation with side walls and over etching and measured results. The two simulated spectra follow the same trend on CIE diagram which indicates that the performance of the two designs is the same.


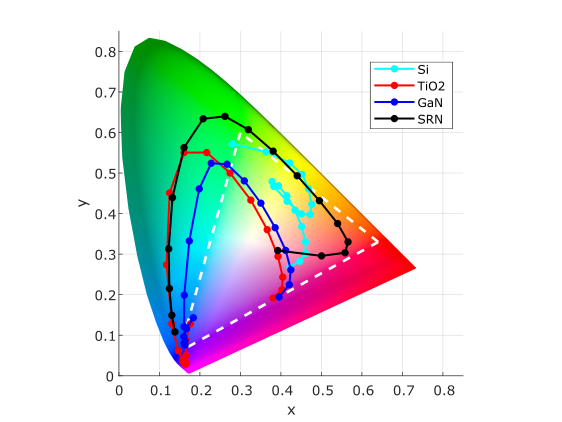


Figure S7: Simulated gamut coverage providing a comparison between silicon, TiO2, GaN and SRN2.99. The parameters used in the simulation are a height of 135nm, radius = 60-150nm with a constant gap of 130nm. We see that SRN2.99 has a wider coverage compared to all the other alternatives. The optical constants for silicon and TiO2 were taken from the simulation bank (Palik and Siefke respectively). For GaN we used the constants from Kawashima.

S8. Translating between reflection spectra and coordinate on the CIE -1931 chromaticity space is obtained via the following equations:

$$X=\frac{1}{K}\int_{\lambda_{0}}^{\lambda_{1}} I\left( \lambda\right)R\left( \lambda\right)\bar{x}\left( \lambda\right)d\lambda$$

$$X=\frac{1}{K}\int_{\lambda_{0}}^{\lambda_{1}} I\left( \lambda\right)R\left( \lambda\right)\bar{y}\left( \lambda\right)d\lambda$$

$$Z=\frac{1}{K}\int_{\lambda_{0}}^{\lambda_{1}} I\left( \lambda\right)R\left( \lambda\right)\bar{z}\left( \lambda\right)d\lambda$$

And K is given by:

$$K=\int_{\lambda_{0}}^{\lambda_{1}} I\left( \lambda\right)\bar{z}\left( \lambda\right)d\lambda$$

The chromaticity coordinates now can be derived by:

$$x=\frac{X}{X+Y+Z}$$

$$y=\frac{Y}{X+Y+Z}$$

S9.

The choice of the metasurface thickness was determined by finding the optimal height which will allow for coverage of the blue region in the bottom left corner of the sRGB region in the CIE diagram.


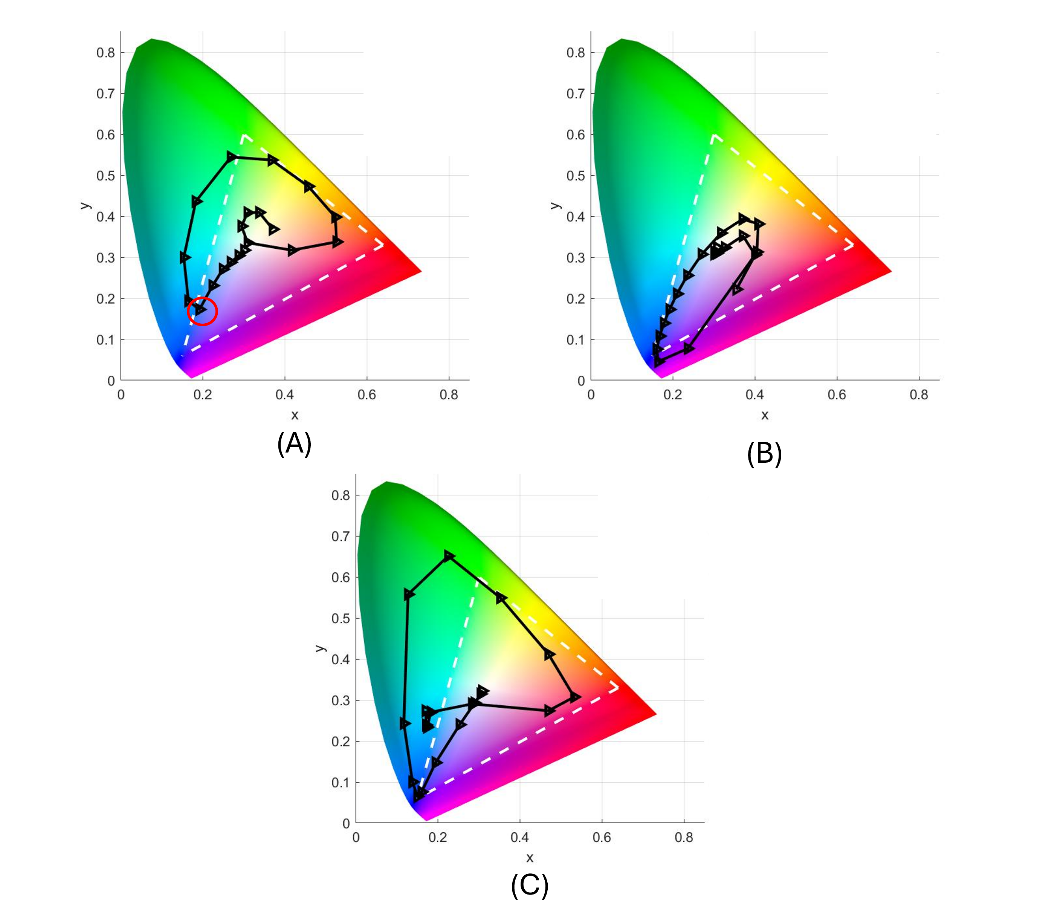


Figure S8:

We first arbitrarily chose the parameters of the unit cell such that the thickness of the disk will be 180nm and the gap between adjacent elements is 130nm for the SRN ratio of $\beta=2.79$. We run a reflection simulation and sweep for different radii values keeping the relation of P=2R+130nm. The results can be seen in Figure S8.A. We then picked the point which is closest to bottom left corner (R=60nm, P=250nm) and swept the thickness of the disk as seen in Figure S8.B. We see that for h=135nm we reach our desired coverage of the blue region. We then repeated the sweep from Figure S8.A and see a great improvement in the gamut coverage.


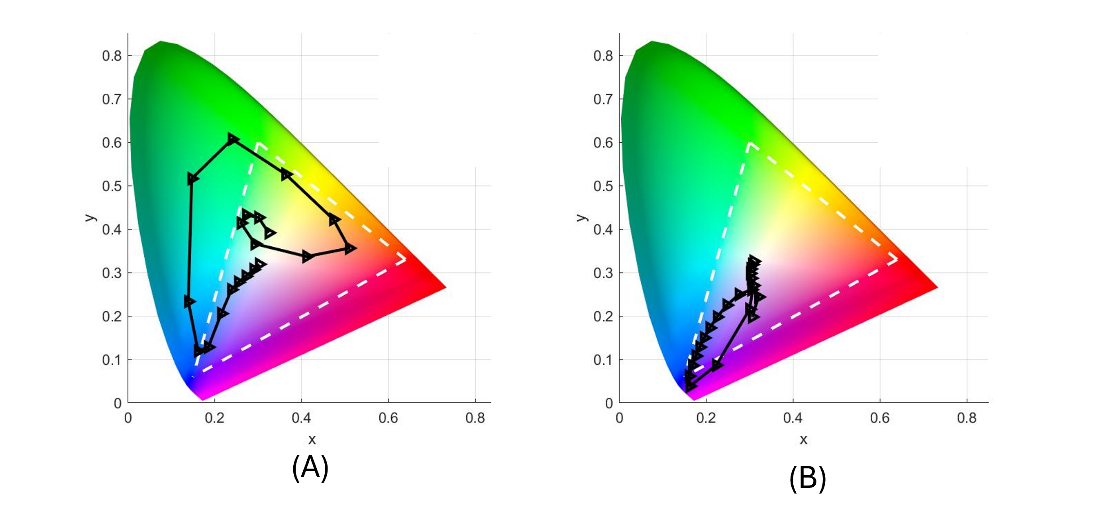


Figure S9:

We repeated the process for SRN2.37 (as seen in Figure S9) and found that we get the same for the thickness of h=135 also in this case.


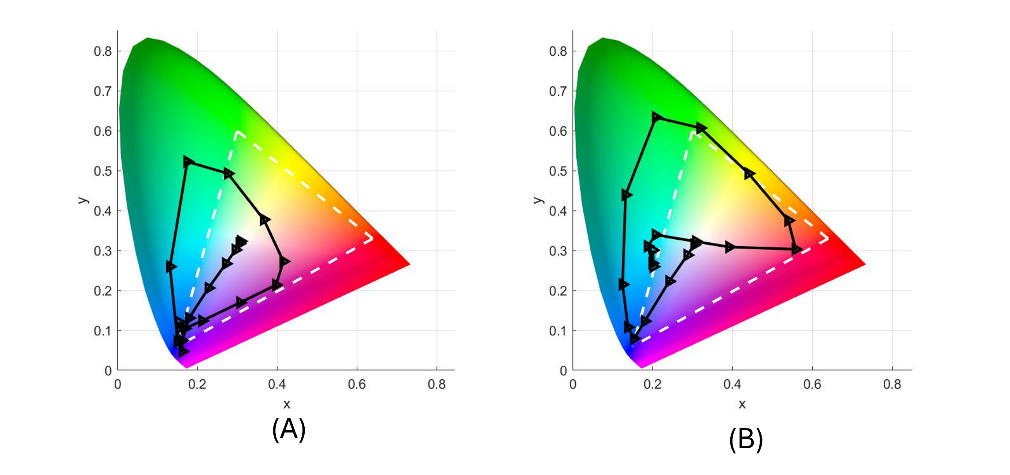


Figure 10

To confirm that this thickness suits our purpose for covering the requested region in the sRGB we repeated the sweep as seen in Figure S8.C for SRN1.77 and SRN2.99 with h=135nm and found that both cover the bottom left corner of the sRGB region as seen in Figure S10. Thus, we found a thickness which allows for all the SRN ratios to cover the wanted point in the CIE diagram.


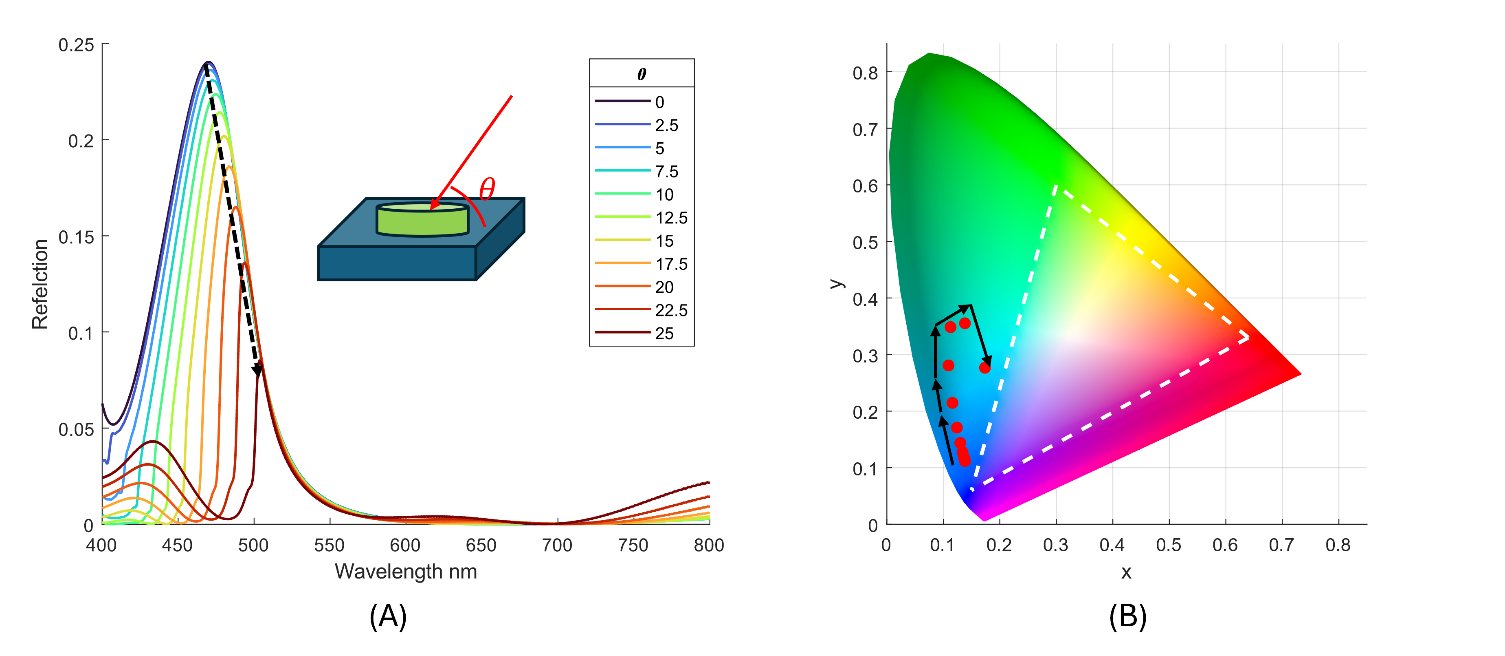


Figure S11: a) Simulated reflection spectra for different illumination angles for a SRN2.99 disk with r=70nm, p=270nm and h=135nm. Increasing the angle of incidence is accompanied by a red shift of the resonance in addition to a decrease in the amplitude. b) CIE diagram for spectra seen in (A). The black arrows indicate the direction in which increase in angle of incidence changes the point on CIE diagram.
